# Commentary: Sulfur Dioxide Contributes to the Cardiac and Mitochondrial Dysfunction in Rats

**DOI:** 10.3389/fcvm.2016.00015

**Published:** 2016-05-26

**Authors:** Salvatore Chirumbolo, Geir Bjørklund

**Affiliations:** ^1^Department of Medicine, University of Verona, Verona, Italy; ^2^Council for Nutritional and Environmental Medicine (CONEM), Mo i Rana, Norway

**Keywords:** mitochondria, heart, sulfur, sulfonated aromatic polymer, sulfonated protein, heart failure

The article by Qin et al. showed that sulfur dioxide contributes in the impairment of cardiac and mitochondrial function in rat H9C2 transformed cardiomyoblasts (1). The authors investigated the effect of 10–100 μM sodium bisulfite (NaHSO_3_) on the mitochondrial function of H2C9 cells and rat primary cardiomyocytes and showed that COX activities, Δψ_m_, and ATP content were decreased by sulfite (1).

This evidence, though restricted to an *in vitro* cell model, should raise a fundamental concern about sulfite preservatives used in wine industry or food and the consequent role of grapes-derived flavonoids in human health. Actually, one of the main sources of sulfites in the human body comes from the addition of sulfites and sulfur dioxide to many food products. The pejorative effect of sulfite preservatives may dampen the beneficial action of wine polyphenols.

Sulfur dioxide dramatically alters grapes transcriptome, leading to mitochondria impairment and downregulation of redox homeostasis (2). Therefore, the presence of sulfur dioxide in plant sources represents an important issue for toxicology, besides any further concern about environmental pollutants. Yet, sulfur dioxide in many conventional, purchasable red wines might not alter necessarily the yield of flavonoids, such as resveratrol, when compared to sulfite-deprived organic wines (3); therefore, its presence or removal should be conventionally reappraised and related to the role than sulfites may have on human health. Yet, toxicology of this food preservative shows many controversial questions in medicine. Although it was considered as an important risk factor for the initiation and progression of liver diseases, based on its proapoptotic and oxidative stressing potential, molecular research on the apoptotic effect of sub-toxicological doses of sulfur dioxide did not appear to fulfill any good expectation, as a controversial debate may occur (4). Figure 1 shows how sulfur dioxide, sulfites, and S-sulfonation of proteins may have different effects on mitochondria and redox signaling. Yet, severe liver injury and necrotic foci were described with the exposure of sulfur dioxide between 56 and 168 mg/m^3^, i.e., within the range 20–60 ppm (5), though far away from the value of 1.5–5.0 ppm, reported by Qin et al. (1). As the same authors addressed in their article, plasma level of sulfites closely depends on dietary habits and the composition of human gut microbiome. Particularly in Western populations, a dietary regimen made by the presence of saturated fatty acids, refined sugars, and low fibers, resembling an experimental milk-derived diet in mice, promotes the expansion of sulfite-reducing bacteria, typically *Clostridia* and other anaerobic species such as *Deltaproteobacteria*, though the profound change in gut microbiome, with increased *Bacteroides*, decreased *Firmicutes* and a marked change in biliary salt chemistry (6). On the other hand, whey retentate, which may come from milk and fermented dairy products (7), promotes the growth of symbiontic *Bifidobacteria* and reduces *Bacteroides fragilis* and sulfite-reducing *Clostridia* (8). Gut microbiome is a formidable barrier for the prevention of sulfur dioxide entering into the plasma and therefore to reach the many target organs, such as myocardial muscle. Furthermore, human plasma levels of [SO_2_]^=^, often in the form of protein-linked *S*-sulfonate, seem to be better correlated with sulfur dioxide in the environmental air, for which an increment of about 1.1 nmol of plasma *S*-sulfonates for any ppm of sulfur dioxide, has been reported (9). This would mean that, besides the clearance activity by human sulfite oxidase (10), sulfur dioxide able to impair cardiovascular function and mitochondrial activity should come quite exclusively from an inner SO_2_ saturated microenvironment, such as in industrial contexts, despite the considerations that most of sulfur would come from diet. Table 1 summarizes some sulfur effects. Although the paper by Qin et al. used an *in vitro* model, their conclusion about human health may be quite premature.

**Figure 1:**
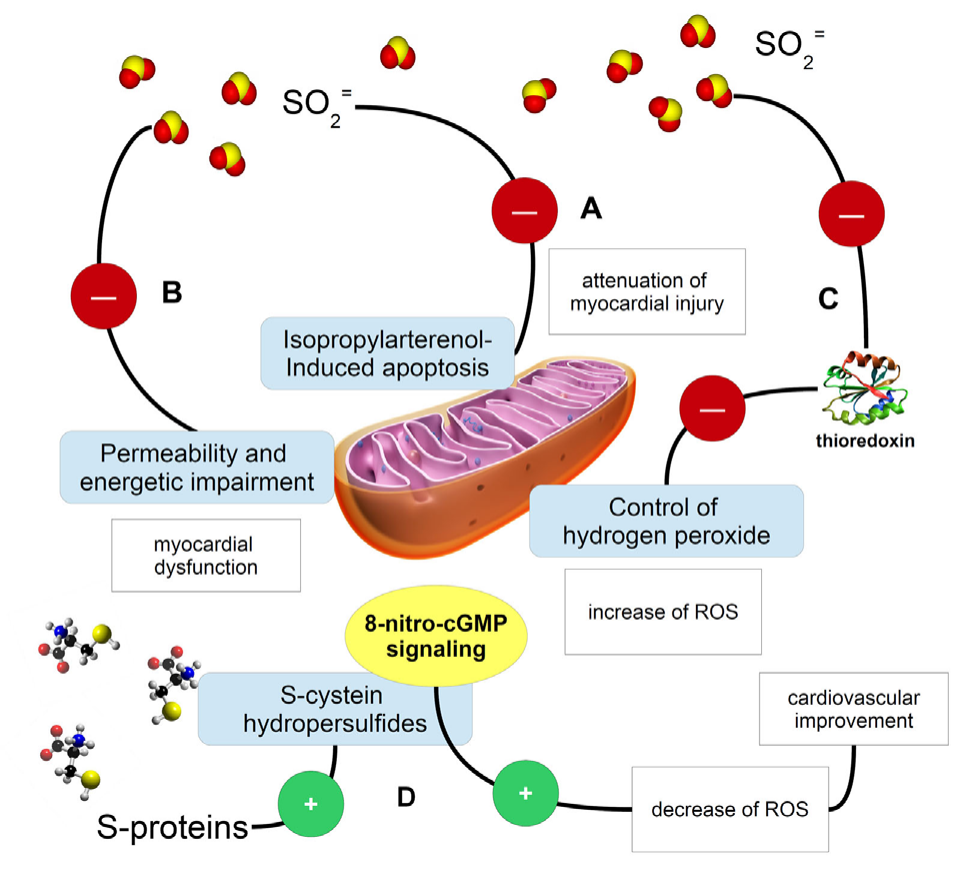
**Cartoon describing some fundamental examples of the activity exerted by sulfur (in the form of sulfur dioxide, sulfites, or S-proteins) on mitochondria and redox signaling**. **(A)** Sulfites and sulfur dioxide are able to inhibit the isopropylarterenol-induced apoptosis and mitochondria dysfunction, so exerting a protective potential on myocardial activity (11); **(B)** the same sulfur-possessing molecules may impair mitochondria permeability and their ability in producing ATP, then causing myocardial dysfunction; **(C)** sulfite ion impairments of mitochondrial function might be explained even with thioredoxin inactivation (12). A mechanistic possibility to explain sulfite ion impairment of mitochondrial function is the evidence that these ions can inactivate thioredoxin (12) and knowing that the thioredoxin 2 system is a major controller of H_2_O_2_ emission from mitochondria (13), sulfite ions can contribute to increased ROS emission from mitochondria that in turn may jeopardize cardiac function; **(D)** *S*-sulfonylated proteins, containing *S*-aminoacids, may be source of reactive sulfur species, such as cysteine hydropersulfides, which are involved in the metabolism of 8-nitroguanosine 3′,5′-cyclic monophosphate. This molecule belongs to the endogenously formed electrophiles, which are important signaling molecules in the ROS and RNS scavenging and regulation (14).

**Table 1:** **Sulfur action on cardiac function and oxidative stress**.

Molecule	Actions/effects	Reference
Sulfur dioxide	Possible long-term cardiac abnormality	Pishgoo et al. (15)
	Cardiac and mitochondrial dysfunction (rats)	Qin et al. (1)
	Increase heart susceptibility to oxidative stress	Zaky et al. (16)
	Inhibits the proapoptotic effect of isopropylarterenol on myocardial tissue through a bcl-2/cytc/caspase-9/caspase-3 pathway	Jin et al. (11)
	Aggravates myocardial I/R injury	Zhang et al. (17)
Sulfur dioxide	Ameliorated systemic hypertension and pulmonary hypertension prevented the development of atherosclerosis and protected against myocardial ischemia-reperfusion (I/R) injury and isoproterenol-induced myocardial injury	Huang et al. (18)
Sulfites		
Sulfur mustard	Modification of the ratio prooxidant/antioxidant	Shohrati et al. (19)
	Reduction of serum GSH, increase of serum malondialdehyde (MDA)	
Sulfites	Deleterious effects through the mitochondrial Fe^3+^ cyt *c*-mediated ROS	Velayutham et al. (20)
	Reduced the length and volume of the ventricular capillaries (rats)	Noorafshan et al. (21)
	With sulfite oxidase contributes in NO signaling	Wang et al. (22)
	They can affect voltage-gated sodium (Na^+^) channels (VGSC) in a concentration-dependent manner in isolated rat ventricular myocytes. Protective action of catechins	Wei and Meng (23)

The authors used a concentration, expressed into micromoles of sodium bisulfite, just approaching the “usual” maximal concentration (≈100 μM) coming from daily intake of food and air pollution, yet the readers are not allowed to comprehend if this apparently “normal” assumption of sulfites may lead to a relevant risk of a cardiovascular damage.

The question is which is the possible meaning of the reported evidence for human health?

Actually, several organic compounds besides sulfites, such as thiosulfates and *S*-sulfocysteine, also coming from biological degradation, may exert a damaging action on mitochondrial permeability transition, mitochondria biogenesis, and energy homeostasis (24). Sulfites themselves then lead to degenerative disorders, e.g., by inhibiting glutamate dehydrogenase in brain (25) or causing impairment in kidney functionality (26). The observed increase in oxidative stress may derive from an impairment in ROS scavenging system, as it is well-known that glutathione *S*-sulfonate, generated from the reaction between glutathione disulfide and sulfite, is a competitive inhibitor of glutathione-*S*-transferase (GST), leading to a marked defect in the detoxification of xenobiotics. In this context, sulfur is strategic in mitochondria-dependent response to oxidative stress, when mitochondrial GST can be activated by direct S-thiolation, while thiol disulfide exchange depends on the correct glutathione redox state. This consideration may suggest that xenobiotics introduced with the diet play a major role, probably much more fundamental than gaseous SO_2_, in regulating mitochondria participation in the homeostasis of oxidative stressors, probably with the involvement of sulfonated-proteins. Actually, an excess of reactive species, also from NO and nitrogen, usually coming from an impairment in the detoxifying machinery, may lead to peroxynitrite-mediated disorder of mitochondrial complex II and peroxynitrite-mediated S-sulfonation, exacerbating the risk of myocardial infarction (27). The paper by Qin et al. showed a marked inhibition of any mitochondrial function by sulfite, probably depending on a different contacting area with cell macromolecules by SO_2_ in the anionic and non-gaseous form.

S-sulfonation is a process by which sulfite interacts with proteins; therefore, it may be a very common mechanism in the biochemistry of human body, possibly causing pathologies, e.g., S-sulfonation of transthyretin has been reported as a trigger step in the formation of amyloid fibrils (28). Protein sulfonation, if evaluated, may give important further insights.

From a toxicological point of view, dietary intake and correct information of gut microbiome should be dealt with the same emphasis of air pollutants, when addressing plant xenobiotics containing sulfur and sulfites present in daily food as preservatives.

## Author Contributions

SC planned, conceived, wrote, and submitted the manuscript; GB prepared the cartoon, searched new references, checked the manuscript, and discussed some important recent points.

## Conflict of Interest Statement

The authors declare that the research was conducted in the absence of any commercial or financial relationships that could be construed as a potential conflict of interest.
